# Conversion therapy with the intent to perform radical local treatment may not be suitable for patients with 10 or more liver metastases from colorectal cancer

**DOI:** 10.1002/cam4.4775

**Published:** 2022-04-25

**Authors:** Junzhong Lin, Hui Sun, Weili Zhang, Zhigang Hong, Zhenhai Lu, Zhizhong Pan, Zhenlin Hou, Jianhong Peng

**Affiliations:** ^1^ Department of Colorectal Surgery Sun Yat‐sen University Cancer Center; State Key Laboratory of Oncology in South China, Collaborative Innovation Center for Cancer Medicine Guangzhou P. R. China; ^2^ Zhongshan School of Medicine Sun Yat‐sen University Guangzhou P. R. China

## Abstract

**Background:**

The number of colorectal cancer liver metastases (CRLMs) is usually considered a contradictory indicator to surgical resection. However, some patients with initially unresectable CRLMs can receive radical local treatment after conversion therapy. This study aimed to evaluate the effect of radical local treatment after conversion therapy and the prognosis of patients with more than 10 initially unresectable CRLMs.

**Methods:**

Data for a total of 229 patients with initially unresectable CRLMs were retrospectively reviewed between December 2012 and January 2020. Among these patients, 107 had ≥10 CRLMs, and 122 had <10 CRLMs. Overall survival (OS) and progression‐free survival (PFS) were used to reflect the prognosis of different groups of patients. Conversion therapy was defined as an initially unresectable liver metastasis converted into an R0 resectable lesion after systemic chemotherapy. Radical local treatment included hepatectomy and radiofrequency ablation (RFA).

**Results:**

Patients with ≥10 CRLMs had a lower conversion rate (42.7% vs. 56.6%, *p* = 0.001). Baseline clinical N stage 1–2, ≥8 first‐line chemotherapy courses, and stable disease (SD) according to the Response Evaluation Criteria in Solid Tumours (RECIST) were independent factors predictive of conversion failure. Primary tumour location in the right colon, RECIST response of SD, and the absence of targeted therapy were independent factors predictive of unfavourable OS. The survival curves revealed that the OS of patients with or without conversion could be distinguished only among patients with <10 CRLMs (89.9% [95% CI, 82.5%–98.0%] vs. 58.9% [95% CI, 45.2%–76.7%], *p* < 0.001); this cut‐off point could also distinguish patients with a successful conversion outcome according to OS (89.9% [95% CI, 82.5–98.0%] vs. 58.2% [95% CI, 42.2–80.4%], *p* = 0.008).

**Conclusion:**

For CRLMs ≥ 10, patients with a successful conversion outcome cannot be distinguished from those without successful conversion outcome according to OS. Thus, conversion therapy with the intent to perform radical local treatment may not be suitable for patients with 10 or more liver metastases from colorectal cancer.

## Graphical abstract

In clinical practice, radical local treatment is still doutble for the primary treatment option for patients with more than 10 initially unresectable liver metastases from colorectal cancer.We find out conversion therapy with the intent to perform radical local treatment may not be suitable for patients with 10 or more liver metastases from colorectal cancer.

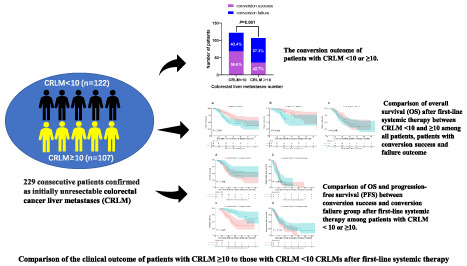

## 1 INTRODUCTION

According to the National Comprehensive Cancer Network (NCCN) clinical practice guidelines,^1^ local treatment is recommended when curative treatment is possible for CRLMs since it can greatly improve overall survival (OS). However, the number of colorectal liver metastases (CRLMs) is usually regarded as a contraindication for hepatectomy.^2^, ^3^ Multiple liver lesions directly reflect a higher tumour burden and further remarkably increase the technical difficulty and feasibility of curative treatment. However, with the increase in tumour response rates due to the popularity of systemic chemotherapy and targeted drugs^4^, ^5^, ^6^, ^7^, ^8^, ^9^, ^10^ and the introduction of ablative technologies,^11^ an increasing number of patients with initially unresectable liver metastases have the chance to achieve curative treatment of CRLM. In current clinical practice, patients with initially unresectable CRLMs are routinely recommended to undergo conversion treatment with the intent of curative treatment.^12^, ^13^, ^14^

During recent decades, the cut‐off value of the number of CRLMs that can be operable has also increased.^2^, ^11^, ^15^, ^16^ In the late 1980s, hepatectomy was considered difficult for patients with more than 3 liver metastases. However, the boundary for the number of liver metastases for resectability reached 5 in 2009.^2^ A recent article proposed that patients with fewer than 10 liver metastases can undergo surgery for removal.^16^ As a result, the current clinical practice guidelines have excluded the tumour number from the constantly evolving criteria.^14^, ^17^

Although previous studies have discussed the role of radical local treatment in the prognosis of patients with a large number of CRLMs, they failed to exclude patients with extrahepatic metastases but instead regarded extrahepatic metastasis only as a risk factor. Moreover, the characteristics and treatment methods of patients with extrahepatic metastases are different from those of patients who have only liver metastases.^18^, ^19^, ^20^ Therefore, it is doubtful that radical local treatment would still be the primary treatment option for patients with more than 10 initially unresectable metastases only in the liver after conversion therapy. This has motivated us to investigate the long‐term survival benefit of these patients receiving conversion therapy. The purpose of this study was to evaluate the prognostic value of radical local treatment after first‐line systemic treatment in patients with at least 10 liver‐only metastases. Additionally, we aimed to determine the prognostic risk factors in these patients.

## 2 METHODS

### 2.1 Study population

We analysed the clinical information of 229 consecutive CRLM patients who were confirmed to have initially unresectable CRLMs from December 2012 to January 2020 at Sun Yat‐sen University Cancer Center. The criteria for selecting the subjects were as follows: (1) histologically diagnosed with colorectal adenocarcinoma, (2) metastases limited to the liver, (3) at least a 3‐month follow‐up period after first‐line systemic treatment, and (4) no previous liver resection or interventional therapy. An electronic medical record system was used to obtain the clinical information and follow‐up results of the patients. Informed consent was obtained from all patients whose clinical data were used. This study was approved by the Institutional Research Ethics Committee of Sun Yat‐sen University Cancer Center (approval number: B2020‐309‐01).

### 2.2 Treatment strategy

The treatment strategy and operability of the liver metastases of each patient were determined according to the final agreement of the multidisciplinary team (MDT), consisting of members from the Departments of Colorectal Surgery, Hepatobiliary Surgery, Medical Oncology, Medical Imaging and Invasive Technology. Tumour response or progression after first‐line treatment was evaluated based on the response evaluation criteria in solid tumours (RECIST).^21^ Conversion therapy was defined as initially unresectable liver metastasis converted to R0 resectable lesion after systemic chemotherapy. Successful conversion outcome refers to initially unresectable liver metastases becoming resectable after first‐line systemic treatment with the patient showing no evidence of disease (NED) owing to radical local treatment, including surgery and radiofrequency ablation (RFA), while conversion failure outcome refers to liver metastases remaining unresectable after first‐line systemic treatment, with the patient unable to receive curative local treatment. Hepatectomy resection was conducted only when the patient met the following criteria^20^: (1) at least one of three liver veins was preserved; (2) more than 30%–40% remnant liver volume was preserved; and (3) the resection margin was at least 1 mm.

### 2.3 Follow‐up

All patients were followed up every 3 months for the first 2 years after hepatectomy and then semi‐annually until 5 years. Follow‐up was conducted by well‐trained nurses. Blood levels of carcinoembryonic antigen (CEA) were measured in each clinical review. Computed tomography (CT) imaging of the chest, abdomen and pelvis was performed at 3, 6, 12, 18 and 24 months and then annually thereafter. Liver magnetic resonance imaging (MRI) was carried out to identify suspicious lesions shown on CT or in cases with negative CT results and elevated levels of CEA. OS was calculated from hepatectomy to death of any cause or the last follow‐up, while progression‐free survival (PFS) was defined as the interval between hepatectomy and recurrence, death or last follow‐up for patients with conversion and between first‐line chemotherapy and disease progression, death or last follow‐up for patients without conversion. The last follow‐up visit took place in April 2021.

### 2.4 Statistical analysis

Statistical analyses were carried out with IBM SPSS Statistics 24 software (IBM), GraphPad Prism version 6.01 (GraphPad Software, Inc.,) and R software packages. Values are presented as the median (range) and percentage. The Kaplan–Meier (K–M) method with the log‐rank test was used to compare PFS and OS. Parameters showing statistical significance in terms of conversion failure, OS and PFS in univariate logistic and Cox models were further analysed by multivariate logistic and Cox models. Odds ratios (ORs), hazard ratios (HRs) and 95% confidence intervals (CIs) were subsequently calculated. All statistical tests used in this study were two‐sided, and a *p* value < 0.05 was considered statistically significant.

## 3 RESULTS

### 3.1 Patient characteristics and systemic therapy

Table 1 presents an overview of the patient characteristics. Among 229 initially unresectable colorectal liver metastasis patients, 165 were males, 64 were female, and the median age was 56 (interquartile range (IQR) 47–62). The median number and IQR of CRLMs for patients with <10 CRLMs and ≥10 CRLMs were 4 (3,6) and 15 (12,27), respectively. All of the patients received first‐line chemotherapy with a median course of 8 (IQR 6–10), and 164 (71.6%) patients received targeted therapy. After first‐line chemotherapy, 112 (48.9%) patients achieved a partial response (PR), 63 (27.5%) patients achieved stable disease (SD), and 54 (23.6%) patients achieved progressive disease (PD). As a result, 105 (45.9%) patients received curative local treatment, including 38 (16.6%) patients who simply underwent hepatectomy and 67 (29.3%) patients who underwent hepatectomy combined with RFA.

**TABLE 1:** Patient demographics, tumour characteristics, and treatment in the total study population

Characteristics	All patients (*N* = 229)	
	No.	%
Patient characteristics		
Age, years, median (IQR)	56 (47–62)	
Sex		
Male	165	72.1
Female	64	27.9
Primary tumour location		
Left colon	109	47.6
Right colon	52	22.7
Rectum	68	29.7
Baseline clinical T stage		
T1–3	122	53.3
T4	107	46.7
Baseline clinical N stage		
N 0	42	18.3
N 1–2	187	81.7
Primary tumour differentiation		
Well to moderate	186	81.2
Poor	43	18.8
Presentation of liver metastases		
Synchronous	210	91.7
Metachronous	19	8.3
Preoperative treatment CEA (ng/mL)		
≤5	60	26.2
>5	169	73.8
RAS status		
Mutation	44	59.4
Wild type	136	19.2
Unknown	49	21.4
Number of liver metastases, median (IQR)	8 (4–15)	
Size of largest liver metastasis (cm),	6 (4–9)	
median (IQR)		
Liver metastases distribution		
Unilobar	48	21.0
Bilobar	181	79.0
Chemotherapy regimen		
Oxaliplatin‐based	147	64.2
Irinotecan‐based	22	9.6
FOLFOXIRI	37	16.2
FUDR HAI	23	10.0
First‐line chemotherapy course, median (IQR)	8 (6–10)	
RECIST response		
PD	54	23.6
SD	63	27.5
PR	112	48.9
Targeted therapy		
No	65	28.4
Bevacizumab	62	44.5
Cetuximab	102	27.1

Abbreviations: CEA, carcinoembryonic antigen; CRLM, colorectal liver metastases; FUDR HAI, fluorodeoxyuridine administered via hepatic arterial infusion; IQR, interquartile range; PD, progressive disease; PR, partial response; RECIST, response evaluation criteria in solid tumours; SD, stable disease.

### 3.2 Comparison of the clinical characteristics of patients with ≥10 CRLMsto those of patients with <10 CRLMs

**FIGURE 1:**
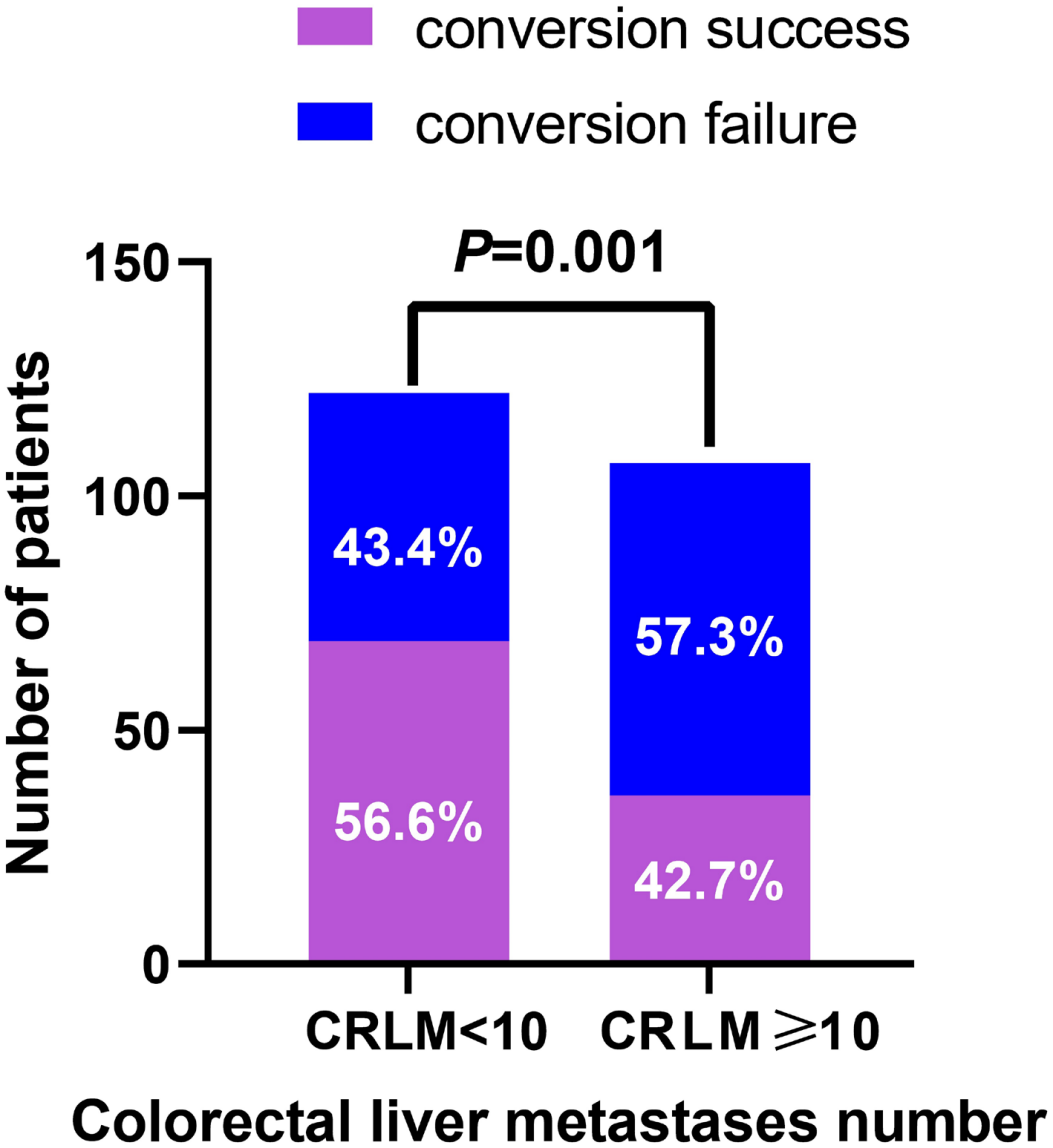
The conversion condition of patients with <10 or ≥10 colorectal cancer liver metastases

As shown in Table 2, compared to patients with <10 CRLMs, a larger proportion of patients with ≥10 CRLMs had synchronous CRLMs (96.3% vs. 87.7% *p* = 0.036), bilobar disease (98.1% vs. 62.3% *p* < 0.001), and PD after systemic therapy (32.7% vs. 15.6%, *p* = 0.006) and underwent more than 8 courses of chemotherapy (46.7% vs. 31.1% *p* = 0.022). In addition, a lower proportion of patients with ≥10 CRLMs had successful conversion outcomes than patients with <10 CRLMs (42.7% vs. 56.6%, *p* = 0.001, Figure 1).

**TABLE 2:** Clinical characteristics of the patients stratified by a cut‐off colorectal cancer liver metastasis number of 10

Characteristics	Liver metastasis number < 10 *n* = 122 (%)	Liver metastasis number ≥ 10 *n* = 107 (%)	*p*value
Age, years			0.219
≤60	75 (61.5)	75 (70.1)	
>60	47 (38.5)	32 (29.9)	
Sex			1.000
Male	88 (72.1)	77 (72.0)	
Female	34 (27.9)	30 (28.0)	
Primary tumour location			0.265
Left colon	64 (52.5)	45 (42.1)	
Right colon	24 (19.7)	28 (26.2)	
Rectum	34 (27.9)	34 (31.8)	
Baseline clinical T stage			1.000
T1–3	65 (53.3)	57 (53.3)	
T4	57 (46.7)	50 (46.7)	
Baseline clinical N stage			0.015
N0	30 (24.6)	12 (11.2)	
N1–2	92 (75.4)	95 (88.8)	
Primary tumour differentiation			0.414
Well to moderate	102 (83.6)	84 (78.5)	
Poor	20 (16.4)	23 (21.5)	
Presentation of liver metastases			0.036
Synchronous	107 (87.7)	103 (96.3)	
Metachronous	15 (12.3)	4 (3.7)	
Preoperative CEA level (ng/ml)			0.095
≤5	38 (31.1)	22 (20.6)	
>5	84 (68.9)	85 (79.4)	
RAS status^a^			1.000
Wild type	75 (75.8)	61 (75.3)	
Mutation	24 (24.2)	20 (24.7)	
Liver metastasis distribution			<0.001
Unilobar	46 (37.7)	2 (1.9)	
Bilobar	76 (62.3)	105 (98.1)	
Chemotherapy regimen			0.240
Oxaliplatin‐based	72 (59.0)	75 (70.1)	
Irinotecan‐based	12 (9.8)	10 (9.3)	
FOLFOXIRI	22 (18)	15 (14.0)	
FUDR HAI	16 (13.1)	7 (6.5)	
First‐line chemotherapy course			0.022
<8 cycle	84 (68.9)	57 (53.3)	
≥8 cycle	38 (31.1)	50 (46.7)	
RECIST response			0.006
PD	19 (15.6)	35 (32.7)	
SD	34 (27.9)	29 (27.1)	
PR	69 (56.6)	43 (40.2)	
Targeted therapy			0.163
NO	35 (28.7)	30 (24.7)	
Bevacizumab	27 (22.1)	35 (32.7)	
Cetuximab	60 (49.2)	42 (39.3)	

Abbreviations: CEA, carcinoembryonic antigen; CRLM, colorectal liver metastasis; FOLFOXIRI, folinic acid, 5‐fluorouracil, oxaliplatin and irinotecan; FUDR HAI, fluorodeoxyuridine administered via hepatic arterial infusion; PD, progressive disease; PR, partial response; RECIST, response evaluation criteria in solid tumours; SD, stable disease.

^a^ Data were available for 180 patients.

### 3.3 Survival outcome

**FIGURE 2:**
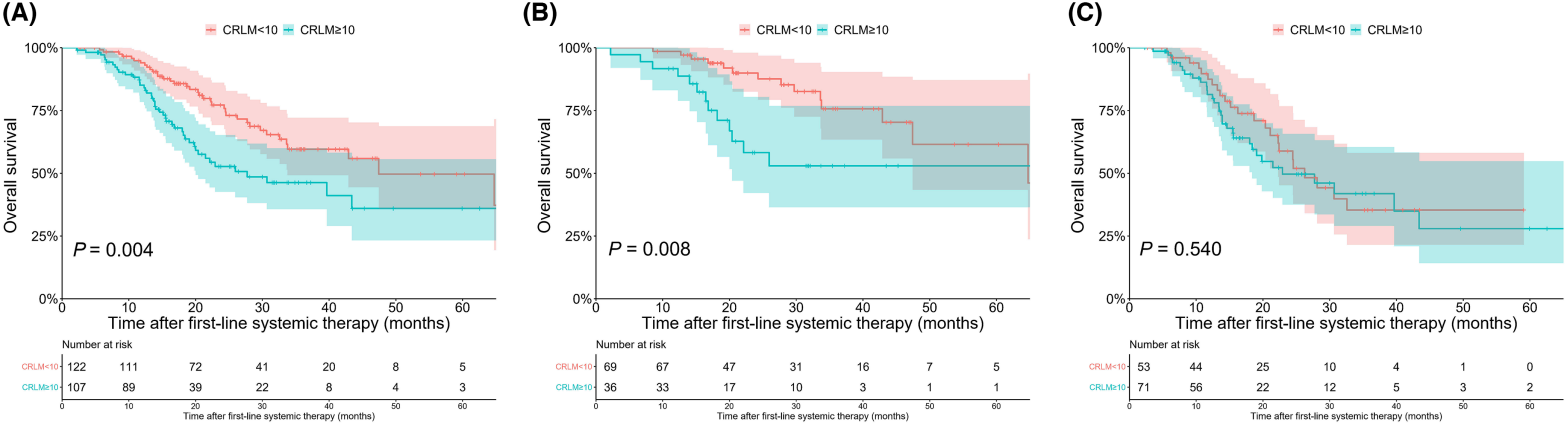
Comparison of overall survival (OS) after first‐line systemic therapy among all patients, patients with conversion success and patients with conversion failure. (A) Comparison of OS in the colorectal cancer liver metastases (CRLM) <10 and CRLM ≥ 10 groups among all patients. (B) Comparison of OS in the CRLM<10 and CRLM≥10 groups among patients with successful conversion outcomes. (C) Comparison of OS in the CRLM<10 and CRLM≥10 groups among patients with failed conversion outcomes

**FIGURE 3:**
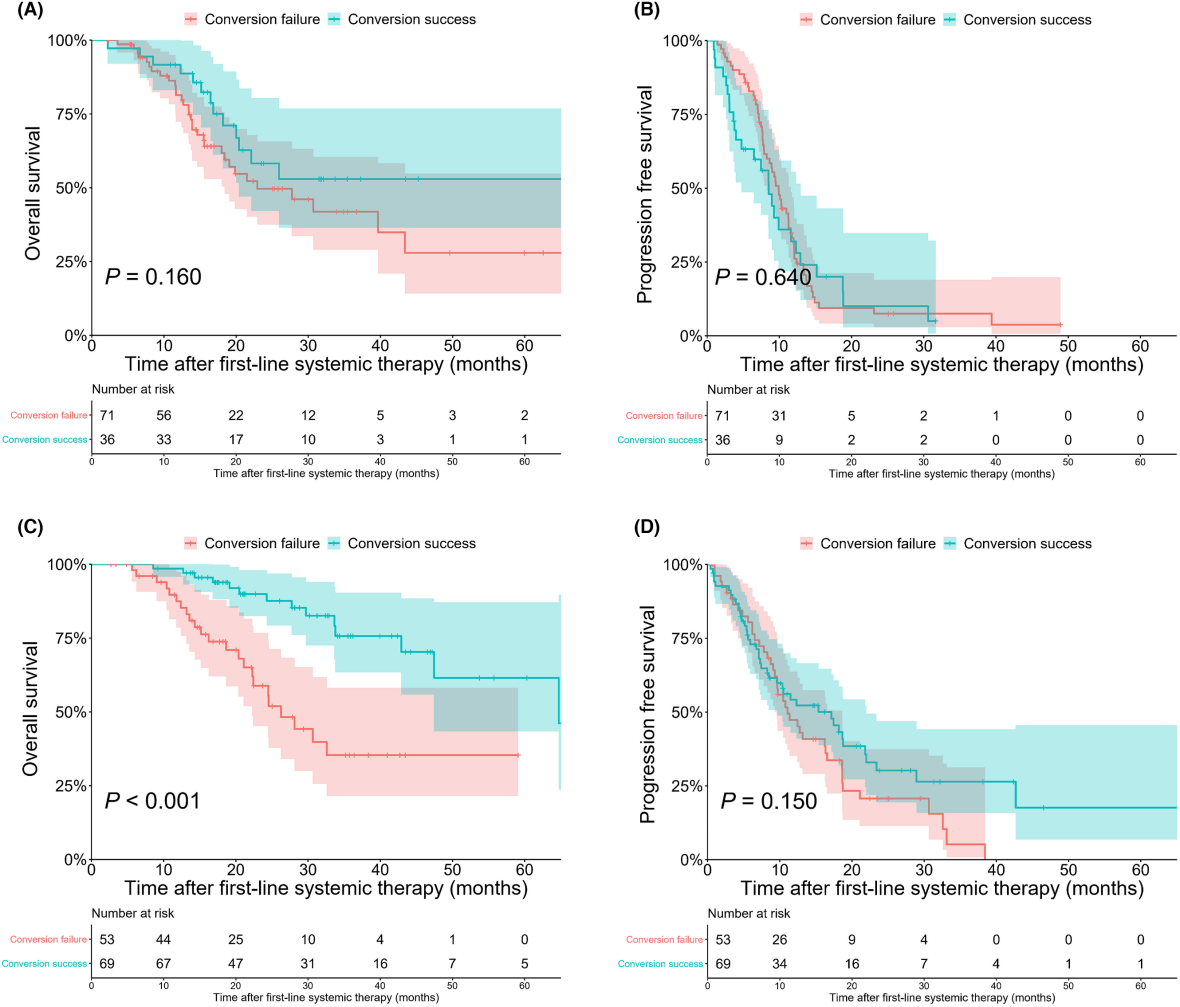
Comparison of overall survival (OS) and progression‐free survival (PFS) after first‐line systemic therapy among patients with <10 or ≥10 colorectal cancer liver metastases (CRLMs). (A) Comparison of OS in the conversion success and conversion failure groups among patients with ≥10 CRLMs. (B) Comparison of PFS in the conversion success and conversion failure groups among patients with ≥10 CRLMs. (C) Comparison of OS in the conversion success and conversion failure groups among patients with <10 CRLMs. (D) Comparison of PFS in the conversion success and conversion failure groups among patients with <10 CRLMs

With a median follow‐up time of 20 months (25%–75% quartiles: 14–32 months), 75 (32.8%) patients were alive with NED, 121 (52.8%) patients were alive with PD, and 33 (14.4%) patients experienced cancer‐related mortality. Patients with <10 CRLMs had significantly higher 2‐year OS rates than those with ≥10 CRLMs [77.2% (95% CI, 69.2–86.0%) vs. 52.7% (95% CI, 42.6–65.2%), *p* = 0.004] (Figure 2A). The univariable and multivariable Cox analyses identified ≥10 CRLMs as an independent predictor of OS (HR 1.629; 95% CI 1.007–2.636; *p* = 0.043) (Table S1). Among the patients with successful conversion outcomes, those with <10 CRLMs showed significantly higher 2‐year OS rates than those with ≥10 CRLMs (89.9% [95% CI, 82.5–98.0%] vs. 58.2% [95% CI, 42.2–80.4%], *P* = 0.008) (Figure 2B). Among patients with conversion failure outcomes, patients with either <10 CRLMs or ≥ 10 CRLMs had similar 2‐year OS rates (58.9% [95% CI, 45.2–76.7%] vs. 49.6% [95% CI, 37.5–65.7%]; *P* = 0.540) (Figure 2C). Among the patients with ≥10 CRLMs, those with successful or failed conversion had comparable 2‐year OS rates (58.2% [95% CI, 42.2–80.4%] vs. 49.6% [95% CI, 37.5–65.7%], *p* = 0.160) (Figure 3A) and PFS rates (7.5% [95% CI, 3.0–18.9%] vs. 10.0% [95% CI, 2.9–34.7%], *p* = 0.640] (Figure 3B). Among the patients with <10 CRLMs, those with a successful conversion outcomes had significantly higher 2‐year OS rates than patients with failed conversion (89.9% [95% CI, 82.5–98.0%] vs. 58.9% [95% CI, 45.2–76.7%], *p* < 0.001) (Figure 3C); however, these two groups of patients had comparable PFS rates (30.2% [95% CI, 19.4–46.9%] vs. 20.7% [95% CI, 11.5–37.4%], *p* = 0.150) (Figure 3D).

### 3.4 Risk factors for conversion failure among patients with ≥10 CRLMs

Table 3 presents the summary statistics for the univariate and multivariate logistic regression analyses. Univariate logistic analysis showed that baseline clinical N stage 1–2 (*p* = 0.016), ≥8 first‐line chemotherapy courses (*p* = 0.002), and SD or PD after systemic treatment (*p* < 0.001) were strongly associated with conversion failure. Multivariate logistic analysis indicated that baseline clinical N stage 1–2 (OR 4.821; 95% CI 1.107–20.990; *p* = 0.036), ≥8 first‐line chemotherapy courses (OR 3.847; 95% CI 1.388–10.665; *p* = 0.010), and a RECIST response of SD or PD (OR 7.408; 95% CI 2.803–19.575; *p* < 0.001) were still independent predictive factors for conversion failure.

**TABLE 3:** Univariate and multivariate logistic regression analyses of risk factors for conversion failure in patients with ≥10 colorectal liver metastases

	Univariable		Multivariable	
Characteristics	OR (95% CI)	*p*value	OR (95% CI)	*p*value
Age (>60 years vs. ≤60 years)	1.437 (0.582–3.548)	0.431		
Sex (male vs. female)	0.981 (0.401–2.398)	0.966		
Primary tumour location (right colon vs. left colon and rectum)	1.373 (0.536–3.516)	0.509		
Baseline clinical T stage (T4 vs. T1–3)	1.616 (0.715–3.654)	0.249		
Baseline clinical N stage (N1–2 vs. N0)	4.786 (1.332–17.190)	0.016	4.821 (1.107–20.990)	0.036
Primary tumour differentiation (poor vs. well to moderate)	2.106 (0.711–6.234)	0.179		
Presentation of liver metastases (synchronous vs. metachronous)	6.364 (0.638–63.517)	0.115		
Preoperative CEA (>5 ng/ml vs. ≤ 5 ng/ml)	1.487 (0.567–3.903)	0.420		
Liver metastases distribution (bilobar vs. unilobar)	2.000 (0.121–32.934)	0.628		
First‐line chemotherapy course (≥ 8 cycles vs. <8 cycles)	4.100 (1.685–9.977)	0.002	3.847 (1.388–10.665)	0.010
RECIST response (SD or PD vs. PR)	8.259 (3.323–20.528)	<0.001	7.408 (2.803–19.575)	<0.001
Targeted therapy (yes vs. no)	0.794 (0.319–1.973)	0.619		

Abbreviations: CEA, carcinoembryonic antigen; CI, confidence interval; OR, odds ratio; PD, progressive disease; PR, partial response; RECIST, response evaluation criteria in solid tumours; SD, stable disease.

### 3.5 Prognostic factors for patients with ≥10 CRLMs

Tables 4 and 5 summarise the univariate and multivariate Cox analyses of OS and PFS for patients with ≥10 CRLMs. Univariate analysis showed that primary tumour location in the right colon (*p* = 0.006), baseline clinical T stage 4 (*p* = 0.005), baseline clinical N stage 1–2 (*p* = 0.041), and a RECIST response of SD or PD (*p* = 0.001) were closely related to unfavourable OS, while the use of targeted therapy (*p* = 0.003) was beneficial to OS. In addition, primary tumour location in the right colon (*p* = 0.028) and SD or PD after systemic treatment (*p* = 0.017) were closely related to unfavourable PFS, while ≥8 first‐line chemotherapy courses (*p* = 0.043) and the use of targeted therapy (*p* = 0.044) were beneficial to PFS.

**TABLE 4:** Univariate and multivariate Cox analyses of risk factors for overall survival in patients with ≥10 colorectal liver metastases

	Univariable		Multivariable	
Characteristics	HR (95% CI)	*p*value	HR (95% CI)	*p*value
Age (>60 years vs. ≤60 years)	1.130 (0.601–2.126)	0.704		
Sex (male vs. female)	0.676 (0.354–1.293)	0.237		
Primary tumour location (right colon vs. left colon and rectum)	2.391 (1.291–4.427)	0.006	2.206 (1.163–4.184)	0.015
Baseline clinical T stage (T4 vs. T1–3)	2.390 (1.309–4.365)	0.005	1.782 (0.933–3.404)	0.080
Baseline clinical N stage (N1–2 vs. N0)	4.411 (1.060–18.356)	0.041	1.888 (0.427–8.353)	0.402
Primary tumour differentiation (poor vs. well to moderate)	1.386 (0.715–2.685)	0.334		
Presentation of liver metastases (synchronous vs. metachronous)	0.621 (0.188–2.050)	0.434		
Preoperative CEA (>5 ng/ml vs. ≤5 ng/ml)	1.446 (0.673–3.110)	0.345		
Live metastasis distribution (bilobar vs. unilobar)	0.311 (0.042–2.297)	0.252		
Conversion outcome (failure vs. success)	1.580 (0.828–3.013)	0.165		
First‐line chemotherapy course (≥ 8 cycle vs. <8 cycle)	0.723 (0.401–1.303)	0.280		
RECIST response (SD or PD vs. PR)	2.706 (1.411–5.188)	0.001	2.053 (1.047–4.023)	0.036
Targeted therapy (yes vs. no)	0.398 (0.218–0.728)	0.003	0.488 (0.263–0.904)	0.022

Abbreviations: CEA, carcinoembryonic antigen; CI, confidence interval; HR, hazard ratio; PD, progressive disease; PR, partial response; RECIST, response evaluation criteria in solid tumours; SD, stable disease.

**TABLE 5:** Univariate and multivariate Cox analyses of risk factors for progression‐free survival in patients with ≥10 colorectal liver metastases

	Univariable		Multivariable	
Characteristics	HR (95% CI)	*p*value	HR (95% CI)	*p*value
Age (>60 years vs. ≤60 years)	0.739 (0.456–1.197)	0.219		
Sex (male vs. female)	0.843 (0.518–1.373)	0.493		
Primary tumour location (right colon vs. left colon and rectum)	1.732 (1.062–2.824)	0.028	1.514 (0.924–2.482)	0.100
Baseline clinical T stage (T4 vs. T1–3)	1.283 (0.836–1.970)	0.254		
Baseline clinical N stage (N1–2 vs. N0)	1.521 (0.755–3.068)	0.240		
Primary tumour differentiation (poor vs. well to moderate)	1.183 (0.715–1.958)	0.513		
Presentation of liver metastases (synchronous vs. metachronous)	1.161 (0.420–3.205)	0.774		
Preoperative CEA (>5 ng/ml vs. ≤ 5 ng/ml)	1.315 (0.769–2.249)	0.317		
Live metastases distribution (bilobar vs. unilobar)	0.465 (0.113–1.911)	0.288		
Conversion outcome (failure vs. success)	0.894 (0.560–1.426)	0.637		
First‐line chemotherapy course (≥ 8 cycle vs. <8 cycle)	0.642 (0.418–0.986)	0.043	0.605 (0.381–0.960)	0.033
RECIST response (SD or PD vs. PR)	1.736 (1.105–2.726)	0.017	1.944 (1.212–3.118)	0.006
Targeted therapy (yes vs. no)	0.612 (0.379–0.988)	0.044	0.693 (0.421–1.140)	0.149

Abbreviations: CEA, carcinoembryonic antigen; CI, confidence interval; HR, hazard ratio; PD, progressive disease; PR, partial response; RECIST, response evaluation criteria in solid tumours; SD, stable disease.

Multivariate analysis revealed that primary tumour location in the right colon (HR 2.206; 95% CI 1.163–4.184; *p* = 0.015) and a RECIST response of SD or PD (HR 2.053; 95% CI 1.047–4.023; *p* = 0.036) were independent predictive factors for unfavourable OS, while the use of targeted therapy (HR 0.488; 95% CI 0.263–0.904; *p* = 0.022) was an independent predictive factor for favourable OS. In addition, SD or PD after systemic treatment (HR 1.944; 95% CI 1.212–3.118; *p* = 0.006) and ≥8 first‐line chemotherapy courses (HR 0.605; 95% CI 0.381–0.960; *p* = 0.033) were independent predictive factors for unfavourable and favourable PFS, respectively.

## 4 DISCUSSION

The number of liver metastases has always been regarded as a risk factor against surgical resection,^2^, ^3^ but with improvements in surgical technology, the introduction of ablative technologies, and the increased popularity of preoperative chemotherapy and targeted drugs, the tumour response rate has been greatly improved, and an increasing number of patients with multiple liver metastases can receive curative treatment.^4^, ^5^, ^6^, ^7^, ^8^, ^9^, ^10^, ^11^ Our data showed that the OS of patients with or without successful conversion outcomes could not be distinguished among patients with ≥10 CRLMs but that the OS of patients with <10 and ≥10 CRLMs could be distinguished among those who had successful conversion outcomes.

The conversion rate of patients with ≥10 CRLMs was significantly lower than that of patients with <10 CRLMs (43.4% vs. 57.3% *p* = 0.001), which was in line with our expectations because patients with a higher number of CRLMs usually progress to the late stage of the disease, and the tumour burden is much higher in these patients than in those with a lower number of CRLMs. To ensure that there is no residual tumour tissue at the surgical margin, patients with ≥10 CRLMs often have more liver volume to be resected; however, liver resection requires a certain proportion of the liver volume to be preserved, so these patients have a lower probability of conversion.^22^ A previous study tried to improve the response rate by various means, including using new drugs, combining different regimens^4^ and introducing targeted therapy,^5^, ^6^ and the conversion rate increased accordingly. Cox multivariate analysis showed that a RECIST response of SD or PD and no use of targeted drugs were significantly associated with poor PFS and OS, suggesting that tumour response is a good predictor of prognosis, which is consistent with the previous studies.^3^, ^14^, ^16^, ^23^

Our results showed that the prognosis of patients with ≥10 CRLMs with successful conversion outcomes was similar to that of patients with failed conversion outcomes, which was inconsistent with the findings of a previous study.^16^ There may be have several explanations for this. First, our patients suffered from initially unresectable liver metastases. As a result, the operation of these patients was still more difficult than that of patients in the previous study, which might result in more postoperative complications.^24^ In addition, patients with ≥10 CRLMs usually have many small nodules that are difficult to detect by CT and MRI.^16^ Even if so‐called R0 or R1 resection is achieved, macroscopic tumours that cannot be detected can recur after resection. Moreover, chemotherapy for patients with ≥10 CRLMs will be more aggressive to meet the operation conditions, which might damage the liver without a nidus. It is worth mentioning that a large number of liver metastatic nodules indicates that the primary tumours are more aggressive, they often have worse pathological types, and some patients do not show good regression after chemotherapy, which leads to a poor prognosis.^25^

From the results of multivariate analysis, whether patients received targeted therapy was an independent prognostic protective factor for patients with >10 metastases. This suggests that targeted therapy should be added when possible to the initial treatment plan for these patients, so effective systemic therapy should be performed in accordance with ESMO or NCCN guidelines.^14^, ^26^ Our findings also reveal that although a large number of metastases is not an absolute no‐go area for NED, local treatment cannot effectively prolong the PFS of patients with >10 metastases, even after conversion therapy achieves resectable criteria, and the survival benefit of these patients is limited. This result demonstrates that local treatment may not be the only option we pursue. We believe that local treatment may be an option for this type of patient, but this needs to be carefully considered in combination with the patient's physical condition, tumour biological behaviour, and treatment willingness. In addition, our study showed that patients with more than 10 metastases and no local treatment had a very high chance of postoperative recurrence, which was accompanied by a worse prognosis. Therefore, the postoperative treatment strategy for such patients should be different from that of patients with <10 CRLMs. For patients with more than 10 CRLMs, adjuvant chemotherapy of sufficient duration and intensity should be ensured after surgery, and more frequent follow‐up should be performed to detect recurrent lesions in time and receive the best treatment as soon as possible.

This study does have some limitations. First, the present study was a single‐centre, retrospective study, which means that further validation of our hypothesis in other institutions is necessary. Second, RAS and BRAF are established prognostic factors; however, we failed to include them in our study due to the insufficient collection of this information. Moreover, the present study analysed only the short‐term outcomes of patients with more than 10 initially unresectable CRLMs, so additional studies focusing on long‐term survival are needed. In addition, since the proportion of patients with ≥10 CRLMs was relatively small, this population is highly selective, and we cannot ensure that identical outcomes would be observed in a less selective patient population.

## 5 CONCLUSION

The OS for patients with and without successful conversion outcome cannot be distinguished among patients with ≥10 CRLMs. Therefore, conversion therapy with the intent to perform radical local treatment may not be suitable for patients with 10 or more liver metastases from colorectal cancer.

## CONFLICT OF INTEREST

None of the authors have conflicts of interest or financial ties to disclose.

## AUTHOR CONTRIBUTIONS

JH Peng, JZ Lin and ZL Hou designed the study; H Sun, WL Zhang, ZG Hong acquired the data; H Sun and WL Zhang conducted the statistical analyses; JH Peng, H Sun and WL Zhang drafted the manuscript. All authors contributed to the interpretation of the results and critical revision of the manuscript for important intellectual content and approved the final version of the manuscript.

## Supporting information


Table S1

## Data Availability

The datasets used and analysed during the current study are available from the corresponding author on reasonable request. The authenticity of this article has been validated by uploading the key raw data onto the Research Data Deposit public platform(www.researchdata.org.cn)with the approval RDD number of RDDA2022862738.
