# Supplementary material for: Conversion therapy with the intent to perform radical local treatment may not be suitable for patients with 10 or more liver metastases from colorectal cancer
Source: Cancer Med. 2022 Apr 25;11(22):4225–35. doi: 10.1002/cam4.4775 (PMC9678090; doi:10.1002/cam4.4775)
Supplement: Supplementary file 1 — Table S1 [file CAM4-11-4225-s001.docx]

**Supplementary Table 1. Univariate and multivariate Cox analyses of risk factors for overall survival in patients with successful conversion outcomes**

|  | **Univariable** | | **Multivariable** | |
| --- | --- | --- | --- | --- |
| **Characteristics** | **HR (95% CI)** | ***P* value** | **HR (95% CI)** | ***P* value** |
| Age (>60 years vs. ≤60 years) | 0.603 (0.268–1.360) | 0.223 |  |  |
| Sex (male vs. female) | 0.683 (0.311–1.502) | 0.343 |  |  |
| Primary tumour location (right colon vs. left colon and rectum) | 1.204 (0.515–2.811) | 0.668 |  |  |
| Baseline clinical T stage (T4 vs. T1–3) | 1.286 (0.624–2.648) | 0.496 |  |  |
| Baseline clinical N stage (N1–2 vs. N0) | 1.639 (0.622–4.315) | 0.318 |  |  |
| Primary tumour differentiation (poor vs. well to moderate) | 1.262 (0.482–3.305) | 0.636 |  |  |
| Presentation of liver metastases (synchronous vs. metachronous) | 0.690 (0.263–1.810) | 0.451 |  |  |
| Preoperative CEA (> 5 ng/mL vs. ≤ 5 ng/mL) | 0.909 (0.433–1.905) | 0.800 |  |  |
| CRLM number (**≥**10 vs.<10) | 3.094 (1.496–6.398) | 0.002 | 3.053 (1.396–6.678) | 0.005 |
| Liver metastasis distribution (bilobar vs. unilobar) | 1.337 (0.571–3.133) | 0.503 |  |  |
| First-line chemotherapy course ($\geq8$cycle vs. <8 cycle) | 0.461 (0.139–1.532) | 0.206 |  |  |
| RECIST response (SD or PD vs. PR) | 1.503 (0.652–3.467) | 0.339 |  |  |
| Targeted therapy  (yes vs. no) | 0.408 (0.188–0.886) | 0.023 | 0.359 (0.165–0.784) | 0.010 |

***HR* hazard ratio, *CI* confidence interval, *CEA* carcinoembryonic antigen, *CRLMs* colorectal cancer liver metastases,** ***RECIST* response evaluation *criteria* in solid tumours, *SD* stable disease, *PD* progressive disease, *PR* partial response**
